# Effect of n-C-S-H on Hydration and Reinforcement of Mineral Powder-Cement System at Low Temperatures

**DOI:** 10.3390/nano14060524

**Published:** 2024-03-15

**Authors:** Wei Li, Chunxiang Qian, Qingchao Li, Kehan Wang, Chunyang Zheng, Yanli Zhang

**Affiliations:** 1 School of Materials Science and Engineering, Southeast University, Nanjing 211189, China; 2 Jiangsu ARIT New Materials Co., Ltd., Nanjing 211505, China

**Keywords:** nano-C-S-H, mineral powder-cement system, hydration mechanism, porosity, early compression strength

## Abstract

This paper investigated the effect of nano-calcium silicate hydrate (n-C-S-H) on the early compressive strength of mineral powder-cement systems under low-temperature curing conditions (5 °C). The hydration mechanism of n-C-S-H in the mineral powder-cement system at different dosages was analyzed by combining it with XRD, DSC-TG, MIP, and other techniques. The results show that n-C-S-H significantly enhances the early compressive strength of the mineral powder-cement system under low-temperature curing conditions, with optimal results observed at a dosage of 1.0% (mass fraction). The XRD, DSC-TG, and MIP tests reveal that n-C-S-H promotes the hydration of the mineral powder cement, accelerates the generation rate of hydration products, reduces the porosity of the hardened mineral powder-cement slurry, and improves the system’s density.

## 1. Introduction

In recent years, the civil engineering and construction industry has shown a strong commitment to environmental friendliness and resource conservation, aligned with the increasing focus on the “dual–carbon” goal. There are numerous studies on the green sustainability of concrete, of which one of the most effective methods is to replace cement with supplementary mortar materials (SCMs), such as fly ash, slag powder, and silica fume [1,2,3]. These materials, which are waste products from other industries, have emerged as sustainable alternatives to cement, significantly reducing the consumption of raw materials, energy, and CO_2_ emissions.

SCMs have become an essential component of concrete. However, the inherent limitations of SCMs prevent them from meeting the requirements of certain projects. Challenges arise, particularly in low-temperature environments and the rapid production of concrete precast products, where the early strength of concrete tends to decrease, making it difficult to meet on-site construction or factory prefabrication specifications. In these cases, it is necessary to add other concrete admixtures, such as accelerators [4,5]. Traditional accelerators have certain drawbacks that are challenging to overcome. For example, chloride-based early strength additives can accelerate the corrosion of steel reinforcement [6], nitrate and sulfate types may negatively impact the late strength and durability of concrete [3,7], while alcohol and amin-based agents have the disadvantages of high costs and dosage sensitivity [1]. Steam curing presents another option, but its high energy consumption can induce internal heat damage, leading to a decline in concrete performance and durability [8,9]. An alternative approach involves the utilization of synthetic nano C-S-H crystal species, which serve as excellent nucleating agents. Their chemical similarity with the main hydration product of cement, C-S-H gel, ensures minimal impact on the pore solution composition within the cement paste, making it an exemplary nucleation matrix for C-S-H gel.

Research has demonstrated that C-S-H can provide more nucleation sites for cement hydration products, lower the nucleation barrier of C-S-H gel, and accelerate the nucleation and growth process of C-S-H gel, thereby enhancing cement hydration and expediting the development of early cement strength [10]. Bost et al. [11] compared the accelerating effects of various early-strengthening agents on cement hydration and revealed that the nano-C-S-H crystalline seed nucleating agent (X-Seed) developed by BASF exhibited a significant effect on both the exothermic quantity and rate of early hydration, leading to a more rapid development of early cement strength [12]. Further, investigations demonstrated that nano-C-S-H also promotes the hydration of fly ash cement. At a 2% dosage of nano-C-S-H, the initial setting time of fly ash cement was reduced to 6.5 h from 8.5 h. When combined with sodium sulfate, the synergistic effect of both further enhanced the early strength of the cement, decreasing the initial setting time to 3.5 h. Furthermore, Nicoleau et al. also observed a significant effect of the nano-C-S-H on the early strength and exothermic rate of cement hydration [13,14]. In an alkali-excited slag system, it was found that nano-C-S-H crystalline species exerted a strong early strength effect, effectively shortening the hydration induction period and increasing the total exothermic heat of hydration. Pedsora et al. [15] examined the influence of nano-C-S-H on the hydration rate of cement at three different temperatures (25 °C, 40 °C, and 60 °C) and found that higher temperatures accelerated the cement hydration process. However, the acceleration effect of n-C-S-H diminished at higher temperatures, and the early strengthening effect tended to decrease in high-temperature environments. The carbonation of concrete occurs during long-term service, thus affecting its durability and strength. The calcium–silicon ratio can accelerate the carbonation process of hydrated calcium silicate, and the calcium–silicon ratio and the C-S-H carbonation rate are inversely proportional, i.e., the larger the calcium–silicon ratio, the lower the C-S-H carbonation rate [16]. Ludwig H M et al. [17] found that nanomaterials generate more C-S-H gels by reacting with silicates, and thus, they exhibit higher durability in terms of resistance to thermal damage, external attacks by acid and sulfate, diffusion of oxides, and surface abrasion, among others, all showing higher durability.

The hydration of cement is not only influenced by the raw materials and their proportions but also exhibits significant dependence on temperature. At lower temperatures, the cement hydration rate decreases, resulting in a slowed strength development that can seriously affect the construction or prefabrication schedules. So far, limited literature has explored the effect of C-S-H nuclei at lower temperatures (e.g., 5 °C) on the reactivity and strength development of SCMS-containing systems. Hence, this study aims to employ n-C-S-H and incorporate a certain proportion of mineral powder to decrease the cement content in the mortar material. The purpose was to tackle the issue of early strength reduction arising from the addition of mineral powder. Additionally, the research seeks to elucidate the mechanism by which n-C-S-H influences the hydration of mineral powder-cement systems through various testing methods, such as XRD, DSC-DTG, MIP, and others. The findings obtained from this study serve as a valuable reference for the practical applications of n-C-S-H in engineering.

## 2. Materials and Methods

### 2.1. Materials

**Figure 1:**
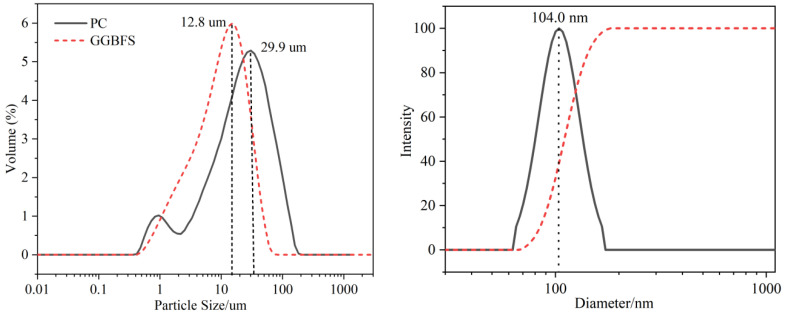
Particle size distribution of PC, GGBFS and n-C-S-H.

In this study, P.I42.5 Portland cement (PC) and GGBFS were used as binders, the chemical composition of the given PC and GGBFS can be found in Table 1, and the particle size distribution curves are shown in Figure 1. ISO standard sand was used for fine aggregate.

The solid content of n-C-S-H solution used in this experiment is 12.1%. The particle size of C-S-H was measured using dynamic light scatterers (Bruker 90 Plus PALS, Karlsruhe, Germany). Before measurement, the n-C-S-H suspension sample was diluted with water to approximately 0.1 g/L, followed by the ultrasonic homogenization of the dispersion for 10 min. The particle size distribution in the n-C-S-H suspension, as obtained from DLS measurements, is shown in Figure 1, the average particle size of it is 104.0 nm.

### 2.2. Methods

#### 2.2.1. Fabrication of Cement Composites

In this experiment, the binder was composed of 60% PC and 40% GGBFS (in mass). The dosage of n-C-S-H was 0, 0.5, 1.0, and 2.0% of the weight of binder. The ratio of water to binder was maintained at 0.35 to prepare samples for subsequent analytical tests (XRD, TG/DSC, etc.).

The effect of n-C-S-H on the properties of the hardened slurry at 5 °C was investigated using a compressive strength test. The mortar prism block (40 × 40 × 160 mm) was prepared according to Table 2 and then cured at 5 °C with a relative humidity of more than 95% for the specific durations (1 d, 3 d, and 28 d). Following the curing period, the specimens were subjected to the compressive strength test.

#### 2.2.2. Characterization

Setting time test: The setting time of the slurry was determined using the Vicat test in accordance with ASTM C191-13 standards [18].

X-ray diffraction (XRD) analysis: XRD analysis was conducted using a Dmax/RB type X-ray diffractometer manufactured by Rigaku Corporation, Tokyo, Japan. The samples, cured to a specified age, were ground into powder, dried, and subjected to testing. The specific test conditions were as follows: Cu-targeted Ka-rays, tube voltage of 40 kV, tube current of 100 mA, diffraction angle of 10–70°, and scanning speed of 5°/min.

The effect of n-C-S-H on the hydration kinetics of PG binder was studied using a TAM-AIR 8-channel microcalorimeter. PG slurry is obtained by mixing PG, water, and n-C-S-H suspension. The water–binder ratio was 0.35, and n-C-S-H was 0, 0.5%, 1.0%, and 2.0% of the mass of PG binder, respectively. The test temperature was 5 °C, and the test duration was 72 h.

Thermogravimetry–differential scanning calorimetry (TG-DSC) analysis: TG-DSC was used to investigate the effect of n-C-S-H on the degree of cement hydration. Prior to the test, the samples were ground into powder and dried in a vacuum freeze dryer for 24 h. During the test, the heating temperature range was 20 °C to 1000 °C, with a heating rate of 20 °C/min. A N_2_ atmosphere was maintained throughout the procedure.

Pore structure test (MIP): The samples that had been cured for the specified durations were cut into 3 mm sized pieces, had their surfaces removed, and were dried in an oven for 24 h. The pore structure and pore size distribution of the samples were assessed using Poremaster GT-6.0 and Quantan chrome mercuric pressure meter (MIP) from the United States.

Strength testing of mortar: The strength test method of mortar prisms was performed according to the procedures outlined in the GB/T 17671-2021 standard, specifically “Strength Test Method of mortar Sand (ISO Method)” [19]. The compressive strength tests of the mortar prisms were carried out at intervals of 1 d, 3 d, and 28 d using a SANS electronic universal testing machine.

## 3. Results and Discussion

### 3.1. The Influence of n-C-S-H on Workability of Cement Paste

**Figure 2:**
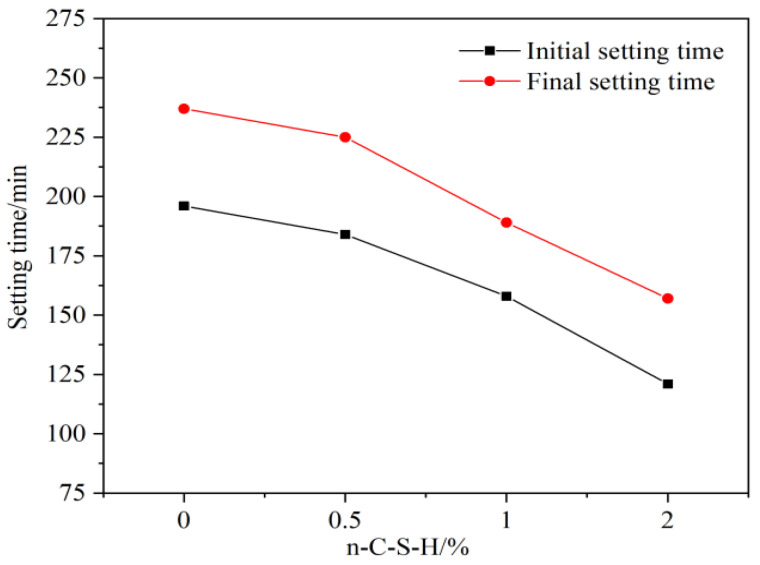
Influence of n-C-S-H on setting time of cement paste.

The results of this study on the effect of varying n-C-S-H dosages on the setting time of cement mortar are shown in Figure 2. It can be seen that the introduction of n-C-S-H effectively reduced the initial setting time of the net cement paste, with a more pronounced effect observed at larger dosages. In the absence of n-C-S-H, the initial setting time of the cement paste was 196 min. Conversely, when n-C-S-H was incorporated at dosages of 0.5%, 1.0%, and 2.0%, the initial setting time was shortened by 6.1%, 19.4%, and 38.3%, respectively. This indicates that the larger the dosage of n-C-S-H, the more significant the effect of promoting the cement setting.

### 3.2. Effects of n-C-S-H on Mechanical Properties of Mortar

**Figure 3:**
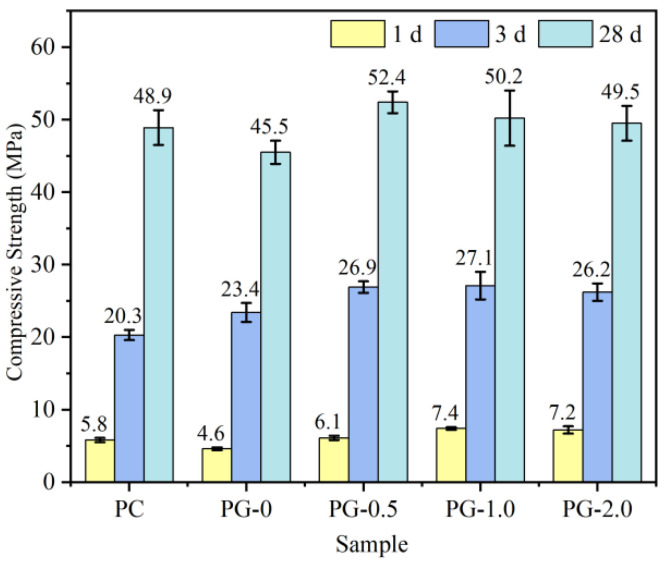
The influence of n-C-S-H on mechanical properties of the mortar.

Figure 3 depicts the impact of n-C-S-H on the compressive strength of the mortar under 5 °C curing conditions. It can be seen that n-C-S-H doping significantly improves the early compressive strength of the mortar at 5 °C. Moreover, with the rise in n-C-S-H doping, the compressive strength of the specimen initially increases and then declines, reaching a maximum at a doping level of 1.0%. At this point, the compressive strength of the 1 d specimen is elevated by 60.9% compared to that of the PG group. Similarly, with a 2.0% n-C-S-H doping, the degree of strength enhancement diminishes, but the specimen’s compressive strength is still 56.5% higher than that of the PG group. In order to better reflect the strengthening effect of n-C-S-H, a GGBFS-free cement component (PC) was added. Compared with the PC group, the compressive strength of the PG-1.0 component increased by 27.6% and 10.3% after 1 day and 28 days, respectively, indicating that the addition of n-C-S-H can compensate for the early activity reduction caused by mineral powder. To further elucidate the enhancement mechanism, the hydration heat test was conducted.

### 3.3. The Effect of n-C-S-H on Cement Hydration

To investigate the enhancement mechanism of n-C-S-H at low temperatures, the effects of n-C-S-H on the hydration rate at different doping levels were independently examined, and the findings were then compared with the differences observed at different initial temperatures.

**Figure 4:**
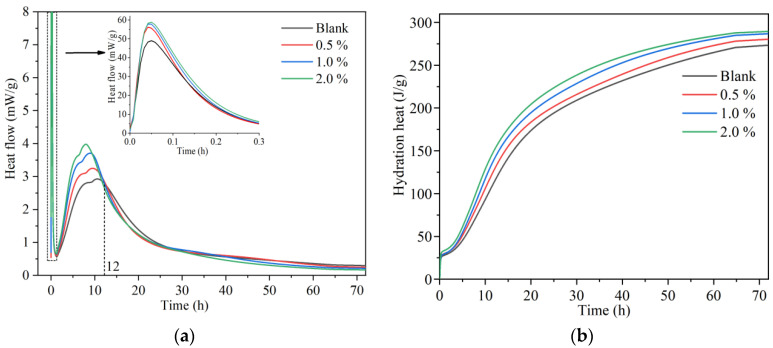
Heat flow curves (**a**) and cumulative heat curves (**b**) of cement paste.

Figure 4 illustrates the influence of different dosages of n-C-S-H on the hydration process of cement paste with a water-to-cement ratio of 0.35. It can be observed that the rate of cement hydration curve shifts closer to the *Y*-axis after the addition of n-C-S-H, with an increased peak. This indicates a significant reduction in the induction period of cement hydration and a gradual increase in the peak heat release rate with a higher n-C-S-H dosage. Combined with the results shown in Table 3, it is evident that n-C-S-H significantly increases the cumulative heat release within the 1-day age of cement, highlighting the promotional effect of n-C-S-H on hydration primarily within the 1 d age, particularly around 12 h. By the 3 d age, the difference in cumulative heat release between the n-C-S-H-incorporated and blank samples diminishes, suggesting a reduced promotion effect of n-C-S-H on cement hydration after 1 d. The gel C-S-H, as the primary hydration product of cement, plays a crucial role in determining the properties of cementitious materials. The growth surface area of C-S-H is determined by the total number of C-S-H nuclei formed during the nucleation process. In comparison to the hydration process of ordinary cement, the hydration products of cement with added n-C-S-H can nucleate and grow not only on the surface of cement particles but also on n-C-S-H seeds. This nucleation method significantly weakens the dissolution barrier of C_3_S in the early stage of cement hydration, shortening the induction period and advancing the acceleration period.

**Figure 5:**
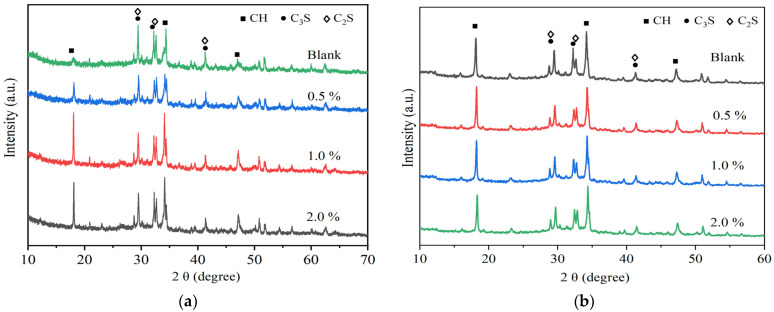
The XRD patterns of the n-C-S-H/cement composites: (**a**) 1 d and (**b**) 28 d.

The formation of the C-S-H phase plays a crucial role in the strength development of cement. Figure 5a shows the effect of n-C-S-H doping changes on the mineral composition of cement. It can be seen that the peak shape and position of each characteristic diffraction peak of the hydration products remained unchanged after the introduction of n-C-S-H into the cement. This indicates that n-C-S-H did not change the type of hydration products but only influenced the intensity of their characteristic diffraction peaks during the early stages of hydration. Notably, the characteristic diffraction peak of CH (2θ = 18.2°) in the early stage of cement hydration (1 d) was significantly enhanced compared to the blank sample. This enhancement suggests that the addition of n-C-S-H effectively promoted the generation of CH as a hydration product during the initial stages of cement hydration. The accelerated rate of cement hydration by n-C-S-H is attributed to the expedited reaction between C-S-H gel and CH, resulting from the enhanced hydration of C_3_S.

As depicted in Figure 5b, in the 28-day sample, the peak intensity of CH in the hydration product of cement doped with n-C-S-H only shows enhancement compared to that of the blank sample. This indicates that the acceleration effect of n-C-S-H on cement hydration is reduced at the age of 28 d. This implies that the primary role of the nucleation sites provided by n-C-S-H is to promote the early-stage generation rate of the C-S-H gel during cement hydration. Since XRD could not be used for quantitative analysis, TG was used to assess the amount of CH production at different ages.

**Figure 6:**
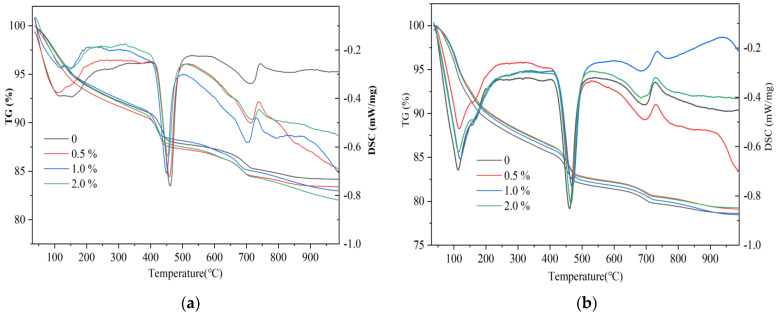
TG/DSC curves after (**a**) 1 d and (**b**) 28 d.

Figure 6 shows the TG-DSC curves of each sample group at 1 d and 28 d. The weight loss below 105 °C corresponds to the removal of non-evaporated water, while the continuous weight loss from 100 °C to 200 °C is attributed to the removal of the weakly bound water in calovanite and C-S-H gel. The noticeable weight loss between 400 °C and 500 °C results from the decomposition and dehydration of CH. Additionally, more pronounced weight loss peaks in the range of 650–750 °C are associated with the decomposition of calcium carbonate (CaCO_3_) generated by the carbonization of the sample during the conservation process [20]. The calcium hydroxide (Ca(OH)_2_, CH) content serves as a measure of the degree of cement hydration and can be determined from the TG curve by using the following equation:

(1) $${\mathrm{Ca}(\mathrm{OH})}_{2}\overset{400–500\;℃}{\to }{\mathrm{CaO}+H}_{2}O↑$$

(2) $${\mathrm{CaCO}}_{3}\overset{650–750\;℃}{\to }{\mathrm{CaO}+\mathrm{CO}}_{2}↑$$

(3) $$m_{\mathrm{CH}}(\%)=\frac{M_{\mathrm{CH}}*m_{H_{2}O}}{M_{H_{2}O}}+\frac{M_{\mathrm{CH}}*m_{{\mathrm{CO}}_{2}}}{M_{{\mathrm{CO}}_{2}}}$$

In the formula, $m_{{\mathrm{CO}}_{2}}$ and $m_{H_{2}O}$ represent the mass loss rate caused by the thermal decomposition of CaCO_3_ and CH, respectively, expressed in percentage. $M_{\mathrm{CH}}$, $M_{H_{2}O}$, and $M_{{\mathrm{CO}}_{2}}$ represent the molecular weights of CH, H_2_O, and CO_2_, respectively.

**Figure 7:**
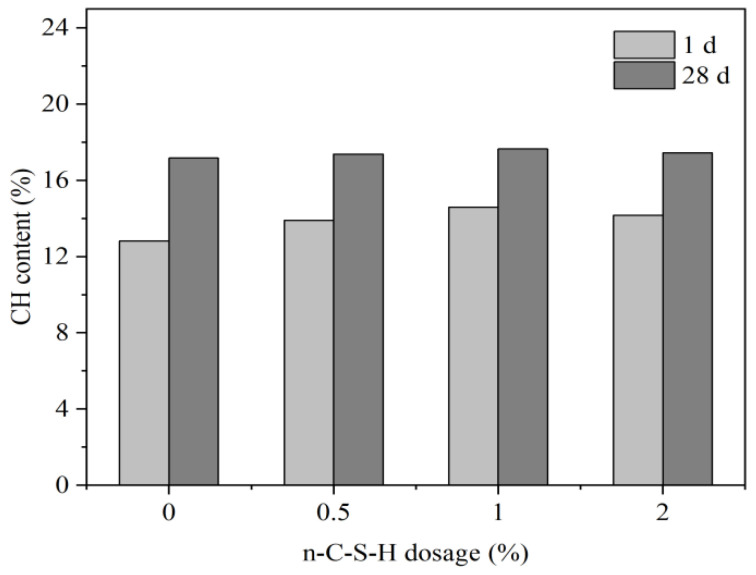
The relationship of the n-C-S-H content and CH concentration.

Figure 7 shows the changes in CH content within cement hydration products after the addition of n-C-S-H, and it can be found that compared with the blank group, an obvious increase in the CH content of the experimental group, especially 1 d old samples, is observed compared to the blank group. This proves that n-C-S-H can accelerate cement hydration and enhance the hydration process. After 28 d, the difference in the CH content between the cement paste doped with n-C-S-H and the blank paste is not significant, indicating that the promotion effect of n-C-S-H on cement hydration gradually decreases in the middle and late stages of hydration. This decline suggests that the nucleation promotion effect of n-C-S-H is mainly reflected in the early stage of cement hydration. Therefore, after 28 d of curing, the gap between the CH content of each group of samples decreases.

### 3.4. Effects of n-C-S-H on the Microstructure of Hardened Cement

**Figure 8:**
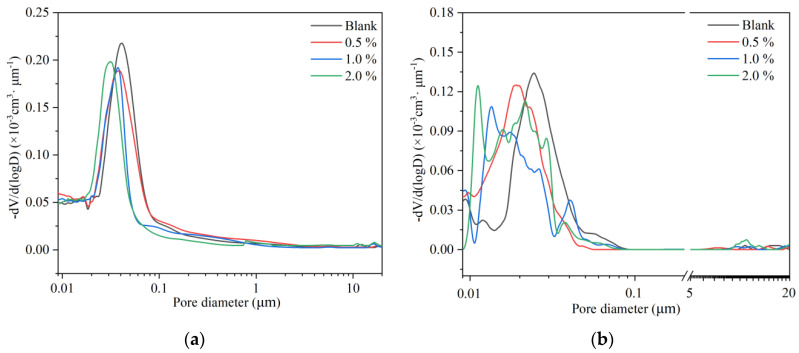
Pore size distribution of the n-C-S-H/cement composites cured: (**a**) pore size distribution at 1 d and (**b**) pore size distribution at 28 d.

Hardened cement paste is a typical porous material, containing pores ranging from nanometer to micrometer and even millimeter scales. Its pore structure significantly influences the mechanical properties, permeability, and durability of hardened mortar materials. Moreover, the strength of hardened cement is inversely proportional to its total porosity [21]. In the case of constant overall porosity, the strength of the cement is inversely proportional to the average pore size [22]. Figure 8 illustrates the impact of n-C-S-H on the pore structure of hardened cement paste, while Table 4 provides comprehensive data on the total porosity and median pore diameter for each group of samples. The overall porosity and the median pore diameter of hardened cement slurry gradually decrease with increasing reaction time, stemming from the continuous generation of hydration products that fill the interstitial spaces between cement particles. Similarly, the overall porosity and the pore content exceeding 50 nm also decrease upon the introduction of n-C-S-H. At a doping level of 1.0%, the total porosity for the 1-day samples is 22.48%, and for the 28-day samples, it is 12.69%, representing reductions of 3.32% and 2.52% compared to the baseline group, respectively. This enhanced densification is attributed to the accelerated hydration of n-C-S-H, which preferentially generates hydration products in the early stages of hydration. Moreover, these hydration products are easier to generate in the capillary pores, facilitating their filling and refinement. Simultaneously, due to its nanoscale nature, n-C-S-H can effectively reduce the porosity by filling the smaller pores, which significantly improves the performance of the cement.

## 4. Conclusions

The incorporation of n-C-S-H improves the strength of mortar. It significantly enhances the early compressive strength of mortar; however, the effect of compressive strength enhancement decreases gradually after 28 d. n-C-S-H effectively addresses the activity gap between the early mineral powder-cement system and the pure cement system.n-C-S-H shortens the induction period of cement hydration and accelerates the overall process. The combination of XRD and TG-DSC results showed that the addition of n-C-S-H did not alter the type of hydration products of mineral powder-cement. However, it does lead to the increased generation of CH hydration products within the initial 24 h. The aforementioned results demonstrate that n-C-S-H significantly promotes the early hydration of cement. Furthermore, the results from the MIP test show a reduction in capillary pore porosity and a decrease in median pore diameter of hardened cement paste containing n-C-S-H. The densification of the pore structure, facilitated by n-C-S-H, significantly improves the performance of the mortar materials. As a nano-crystalline-nucleating early-strengthening agent, n-C-S-H offers a valuable reference for the design of the composite mortar material system with a high proportion of low-activity mineral admixture and theoretical research.

## Figures and Tables

**Table 1:** Chemical composition of Portland cement (wt%).

Composition	Percentage/%
CaO	64.42
SiO_2_	21.72
Al_2_O_3_	4.52
Fe_2_O_3_	3.16
SO_3_	0.56
MgO	1.64
K_2_O	0.69
Na_2_O	0.16
P_2_O_5_	0.14
MnO	0.10
LOI	1.82

**Table 2:** Mix proportion of mortar with different dosages of n-C-S-H.

Sample	Cement (g)	GGBFS(g)	Standard Sand (g)	Water (mL)	Dosage of n-C-S-H (w/%)
PG-0	270	180	1350	180.0	0
PG-0.5	270	180	1350	178.0	0.5
PG-1.0	270	180	1350	176.0	1.0
PG-2.0	270	180	1350	172.1	2.0

**Table 3:** Cumulative heat release of PG slurry in different time periods (J/g).

n-C-S-H (%)	12 h	24 h	72 h
0	114	190	273
0.5	128	199	280
1.0	140	211	286
2.0	151	220	289

**Table 4:** Effects of n-C-S-H on cement porosity and median pore diameter.

n-C-S-H (%)	Porosity (%)		Median Pore Diameter (nm)	
	1 d	28 d	1 d	28 d
0	25.80	15.21	35	26
0.5	26.16	15.11	32	21
1.0	22.48	12.69	30	19
2.0	26.64	14.86	27	21

## Data Availability

The data presented in this study are available on request from the corresponding author.

## References

[1] Hoang K., Justnes H., Geiker M. (2016). Early age strength increase of fly ash blended cement by a ternary hardening accelerating admixture. Cem. Concr. Res..

[2] Juenger M.C.G., Siddique R. (2015). Recent advances in understanding the role of supplementary mortar materials in concrete. Cem. Concr. Res..

[3] Li Q., Zhang L., Gao X., Zhang J. (2020). Effect of pulverized fuel ash, ground granulated blast-furnace slag and CO_2_ curing on performance of magnesium oxysulfate cement. Constr. Build. Mater..

[4] Wu M., Zhang Y., Ji Y., Liu G., Liu C., She W., Sun W. (2018). Reducing environmental impacts and carbon emissions: Study of effects of superfine cement particles on blended cement containing high volume mineral admixtures. J. Clean. Prod..

[5] He J., Long G., Ma K., Xie Y. (2021). Influence of fly ash or slag on nucleation and growth of early hydration of cement. Thermochim. Acta.

[6] Riding K., Silva D.A., Scrivener K. (2010). Early age strength enhancement of blended cement systems by CaCl_2_ and diethanol-isopropanolamine. Cem. Concr. Res..

[7] Ramachandran V.S. (1973). Action of triethanolamine on the hydration of tricalcium aluminate. Cem. Concr. Res..

[8] Mehdipour I., Kumar A., Khayat K.H. (2017). Rheology, hydration, and strength evolution of interground limestone cement containing PCE dispersant and high volume supplementary mortar materials. Mater. Des..

[9] Lothenbach B., Winnefeld F., Alder C., Wieland E., Lunk P. (2007). Effect of temperature on the pore solution, microstructure and hydration products of Portland cement pastes. Cem. Concr. Res..

[10] Liu L., Sun C., Geng G., Feng P., Li J., Dähn R. (2019). Influence of decalcification on structural and mechanical properties of synthetic calcium silicate hydrate (C-S-H). Cem. Concr. Res..

[11] Bost P., Regnier M., Horgnies M. (2016). Comparison of the accelerating effect of various additions on the early hydration of Portland cement. Constr. Build. Mater..

[12] Sun J., Shi H., Qian B., Xu Z., Li W., Shen X. (2017). Effects of synthetic C-S-H/PCE nanocomposites on early cement hydration. Constr. Build. Mater..

[13] Nicoleau L. (2011). Accelerated growth of calcium silicate hydrates: Experiments and simulations. Cem. Concr. Res..

[14] Nicoleau L., Nonat A. (2012). A reply to the discussion “Accelerated growth of calcium silicate hydrates: Experiments and simulations” by S. Bishnoi and K. Scrivener. Cem. Concr. Res..

[15] Pedrosa H.C., Reales O.M., Reis V.D., das Dores Paiva M., Fairbairn E.M.R. (2020). Hydration of Portland cement accelerated by C-S-H seeds at different temperatures. Cem. Concr. Res..

[16] Fu H., Tian L., Wang P., Zuo W., Zhao T., Han X. (2023). Microstructure, deformation and durability of high-strength non-steam-cured concrete with CSH seed. Constr. Build. Mater..

[17] Ludwig H.M., Dressel D. (2011). Synthetische calcium-silikat-hydrate in fertigteilbetonen. BWI.

[18] (2018). Standard Test Methods for Time of Setting of Hydraulic Cement by Vicat Needle.

[19] Standardization Administration of China (2021). Method of Testing Cements-Determination of Strength: GB/T 17671-2021 [S].

[20] Fares H., Remond S., Noumowe A., Cousture A. (2010). High temperature behaviour of self-consolidating concrete: Microstructure and physicochemical properties. Cem. Concr. Res..

[21] Das B.B., Kondraivendhan B. (2012). Implication of pore size distribution parameters on compressive strength, permeability and hydraulic diffusivity of concrete. Constr. Build. Mater..

[22] Kumar R., Bhattacharjee B. (2003). Porosity, pore size distribution and in situ strength of concrete. Cem. Concr. Res..

